# MetaboAge DB: a repository of known ageing-related changes in the human metabolome

**DOI:** 10.1007/s10522-020-09892-w

**Published:** 2020-08-12

**Authors:** Teodora Bucaciuc Mracica, Anca Anghel, Catalin Florentin Ion, Corina Violeta Moraru, Robi Tacutu, Gligor Andrei Lazar

**Affiliations:** 1 Systems Biology of Aging Group, Institute of Biochemistry, Romanian Academy, Bucharest, Romania; 2 Reverse Senescence Biotechnologies SRL, Campia Turzii, Romania; 3 Chronos Biosystems SRL, Bucharest, Romania

**Keywords:** Metabolomics, Ageing, Database, Metabolite variation

## Abstract

Accumulating metabolomics data is starting to become extremely useful in understanding the ageing process, by providing a snapshot into the metabolic state of tissues and organs, at different ages. Molecular studies of such metabolic variations during “normal” ageing can hence guide lifestyle changes and/or medical interventions aimed at improving healthspan and perhaps even lifespan. In this work, we present MetaboAge, a freely accessible database which hosts ageing-related metabolite changes, occurring in healthy individuals. Data is automatically filtered and then manually curated from scientific articles reporting statistically significant associations of human metabolite variations or correlations with ageing. Up to date, MetaboAge contains 408 metabolites annotated with their biological and chemical information, and more than 1515 ageing-related variations, graphically represented on the website grouped by validation methods, sex and age-groups. The MetaboAge database aims to continually structure the expanding information from the field of metabolomics in relation to ageing, thus making it more accessible for further research in gerontology.

**Electronic supplementary material:**

The online version of this article (doi:10.1007/s10522-020-09892-w) contains supplementary material, which is available to authorized users.

## Introduction

Due to medical improvements, the average lifespan within the human population has significantly increased in the last few centuries, revealing new major biomedical challenges which include tackling the incidence of a wide-range of chronic conditions with debilitating effects for the elderly population. Although the manifestations of these age-related pathologies are different, one important common denominator is an exponential increase in prevalence with age (Belikov 2019). Moreover, extensive molecular links have also been shown between ageing and age-related diseases, including cancer, cardiovascular diseases, Alzheimer’s disease, and type 2 diabetes (Fernandes et al. 2016; Tacutu et al. 2011). As a consequence, it is becoming increasingly important for our society to study the biological mechanisms of human ageing in order to complement medical or geriatric solutions.

Unfortunately, fully understanding the gradual decline in physiological functions characterizing the ageing processes in multicellular, eukaryotic systems, requires complex, multi-omics analyses as ageing dysregulations can be found at various levels of organization, from molecules and cells to the entire organism (Lorusso et al. 2018). While current attempts to gain insights into the ageing phenomenon still face limitations and challenges regarding the integration and interpretation of ‘big data’ (Lorusso et al. 2018), recent advancements in high-throughput technologies have resulted in a continuously accumulating amount of data.

Broadly speaking, age-related molecular modifications occur as a consequence of various pathways being altered with age in a cumulative manner. Subsequently, this results in a diverse age-related metabolite pool, which includes among others, products of oxidative degradation and/or secondary products of enzymatic reactions (Tan et al. 2018; Scheibye-Knudsen et al. 2015). As such, metabolomics is beginning to be an extremely useful tool in better understanding the ageing signature, since the quantification of metabolites not only reflects indirectly the variations seen at transcriptional and proteomic levels, but can also offer an instant snapshot of the entire cellular environment (Hoffman et al. 2017; Srivastava 2019). Furthermore, although the ageing-associated metabolic pattern may vary in humans in an organ- or even tissue-dependent manner, and the sexual dimorphism in certain metabolite levels has to be taken into account in most cases, there are metabolites or sets of metabolites that strongly correlate with ageing in multiple organs, independent of gender (Lorusso et al. 2018). A set of metabolites, rather than a single one, can serve as a biomarker for aging. A recent study (Mamoshina et al. 2018), using deep machine learning to predict chronological age based on hematological “aging clocks”, showed that more accurate predictions can be achieved by training these aging clocks on population-specific data or on combined datasets. This optimization can be explained in part by the existence of slightly different aging phenotypes within various ethnic groups, due to a series of factors, including the variability in lifestyle, environmental exposure, diet etc. Lastly, due to their versatility, metabolomics approaches could prove highly useful overall in evaluating lifestyle changes and/or medical interventions aimed at improving health in the elderly (Eline Slagboom et al. 2018), or in a clinical setting in disease aetiology, diagnostic stratification and even therapeutic solutions (Collino et al. 2013).

Because the research of ageing is a rapidly emerging field, new tools for the collection and integration of ageing and age-related disease data are being frequently developed (Hühne et al. 2014). However, according to the JenAge information center (http://info-centre.jenage.de), from the 21 ageing-related databases none is specialized in ageing metabolomics. Here we present MetaboAge, a database and website (http://www.metaboage.info), which hosts manually collected and curated data about age-related metabolic changes from studies of disease-free human cohorts. Through this publicly available resource, we aim to offer the scientific and medical community a general reference source that integrates metabolomics data with additional information from literature sources, and by doing so, to facilitate a better understanding of the metabolic dysregulations that occur in ageing and age-related pathologies.

## Results

### Database design and contents

MetaboAge is a comprehensive database with quantitative, age-related information about metabolite changes in healthy ageing. Ageing-sensitive metabolites are presented with their chemical information, reported variations between age groups, and with the metabolic pathways in which they are involved. Newly added metabolite entries are first annotated automatically and then manually reviewed. Biological and chemical information (such as sample localization, general metabolite documentation, chemical formula, chemical mass, etc.) for each metabolite are extracted from well known databases, such as PubChem, the Human Metabolome Database, ChEBI, ChemIDplus and others (for a full list of considered data sources please see Supplementary Table S1), as well as from the scientific literature. In each case, the automatically retrieved information is carefully revised and manually validated in MetaboAge. It is important to note that MetaboAge distinguishes between protonated and deprotonated compound forms, and metabolites are annotated accordingly. For those metabolites which are not found in any of the common databases, MetaboAge adds a short description based on the article from which those metabolite variations were extracted, including general information but also their effect on ageing and in which gender(s) this effect can be seen.

Besides the general biological and chemical information about each metabolite, MetaboAge provides the users with specific age-related data. The data about metabolite variation within different ageing groups is collected together with the units of measurement, the method by which the metabolite is detected, as well as the age range and sex of each experimental group, and added to MetaboAge. Information on reported locations or sources of the biological sample in which measurements were carried out, from each processed article, are also included in the database and an ontology browser allows users to surf the data based on metabolite locations in the human body.

Lastly, our database uses data from the KEGG database to deliver information about specific pathways that the metabolites are involved in. Various metabolite IDs, linking the MetaboAge entry to each of the used databases, are collected and provide users with easy to use external links.

### Database statistics and overview

The database contains information for more than 400 metabolites, an ontology with 111 classes, and more than 1515 age-related metabolite variations for men and/or women, extracted from 72 scientific articles (at the time of writing). More details on statistics can be seen in Table 1.

**Table 1:** MetaboAge statistics

Metabolite statistics	
Number of metabolites in the database	408
Number of articles for metabolite annotations	72
Number of detection methods for metabolites	62
Metabolite variations statistics	
Number of variations in both genders	673
Number of variations in women	450
Number of variations in men	396
Total metabolite variations	1519
Number of articles for metabolite variations	74

In order to have an overview of the most supported and consistent changes included in MetaboAge, the metabolites in the database were sorted by the number of references from which ageing-related variations were extracted. Based on our entries, lactate and creatine seem to have been the most studied until now in healthy individuals, with 12 and 22 variations, from a total of 6 and 7 scientific articles, respectively. The variations in lactate seem to positively correlate with age across studies, with only one study showing a decrease in its concentration in the older age group. This observation is in accordance with the literature, as it was previously shown that lactate is associated with normal ageing as a consequence of reduced glucose metabolism driven by the loss of aerobic glycolysis (Goyal et al. 2017).

On the other hand, the variation of creatine with age is more complex, with both increases and decreases being reported with age. Still, the number of cases in which creatine levels are higher in the older groups is more than twice as high as the cases in which the metabolite shows higher levels in the younger age groups. Several studies have indicated that disturbances of the creatine, phosphocreatine, and creatine kinase system, are associated with ageing, suggesting that supplementation with creatine can reverse age-associated functional declines (Sumien et al. 2018), however more studies are probably needed to conclude whether this is universal or varies for different populations.

Another metabolite that has been intensely studied with regards to ageing is N-acetyl aspartate, which presents 26 ageing-related variations in MetaboAge (from 8 papers). Similar to creatine, it seems that differences in gender, detection method and sample source influence whether the level of N-acetyl aspartate is rising or falling with age. Lower N-acetyl aspartate serum levels observed in older groups might be explained by a defective activity of N-acetyl aspartate transporters from neurons to oligodendrocytes and/or a compromised N-acetyl aspartate astrocyte uptake (Ruggieri et al. 2011). Also, alterations in the peripheral N-acetyl aspartate physiology (metabolism, excretion, protein binding) have been reported to appear due to ageing (Ruggieri et al. 2011).

Overall, it seems that a meta-analysis of metabolite changes with age is not very straight forward at this point, and the levels of the most studied metabolites can be influenced by many confounding factors, including gender, methodology and prelevation sources (Nakamura et al. 2008). Obviously, for metabolites with variations reported in a moderate number of studies, the story becomes slightly simpler (although it cannot be excluded that a similar diversity will become apparent later). For example, for myo-inositol, ornithine, dehydroepiandrosterone sulfate (DHEA(S)), alpha-tocopherol, hippurate or isoleucine, the observed variations correlate either positively or negatively with age, regardless of method or sample type. Indeed, these correlations with age (either positive or negative) are supported also by other ageing-related studies. Myo-inositol, which is an important osmoregulator in various organs and tissues, showed decreased levels in the human brain in older people compared to younger age groups (Ogawa et al. 1994). Higher concentrations with age of the metabolite ornithine were also reported in acid hydrolyzates of human skin collagen and lens crystallins (Sell et al. 2005). DHEA(S), which is characterized by a low value of blood DHEA(S) with maintenance of cortisol level, was linked to a significant age-related decrease in blood (Baulieu et al. 2000). Isoleucine, which belongs to the branched-chain amino acids (BCAAs) is both an essential amino acid for humans and a signaling molecule. The BCAAs are part of the mechanistic target of rapamycin (mTOR) signaling pathway and are associated with healthy aging. Furthermore, their interaction on a frailty score study has been shown to be statistically significant in a subgroup of older individuals (Zhang et al. 2020). The most bioavailable form of vitamin E, alpha-tocopherol, is an antioxidant fat-soluble compound that appears to influence cognitive function in the elderly. A study focusing on an old population determined that low serum alpha-tocopherol levels negatively affected their cognition capacity, suggesting that there might be a link between vitamin E and mental comprehension in the elderly (Ortega et al. 2002).

While the scope of this paper is not to perform a full analysis of the consistency of age-related changes across metabolomics studies, we believe that MetaboAge can be used as a tool to identify some of the more universal changes and perhaps prioritize those metabolites for which further in vivo assays are still needed to clarify the inconsistency in their changes with age. This guiding process should become even more informative, the more metabolomics studies will be carried out and included in the database.

### User interface

MetaboAge can be accessed at http://www.metaboage.info through an easy and user-friendly web-interface. Users can browse the database either via a list of metabolites, pathways or through a location-based ontology. The database is easily searchable and provides the necessary tools for comparing and downloading age-related metabolomic data in humans, together with chemical, biological and ontology information.

**Fig. 1:**
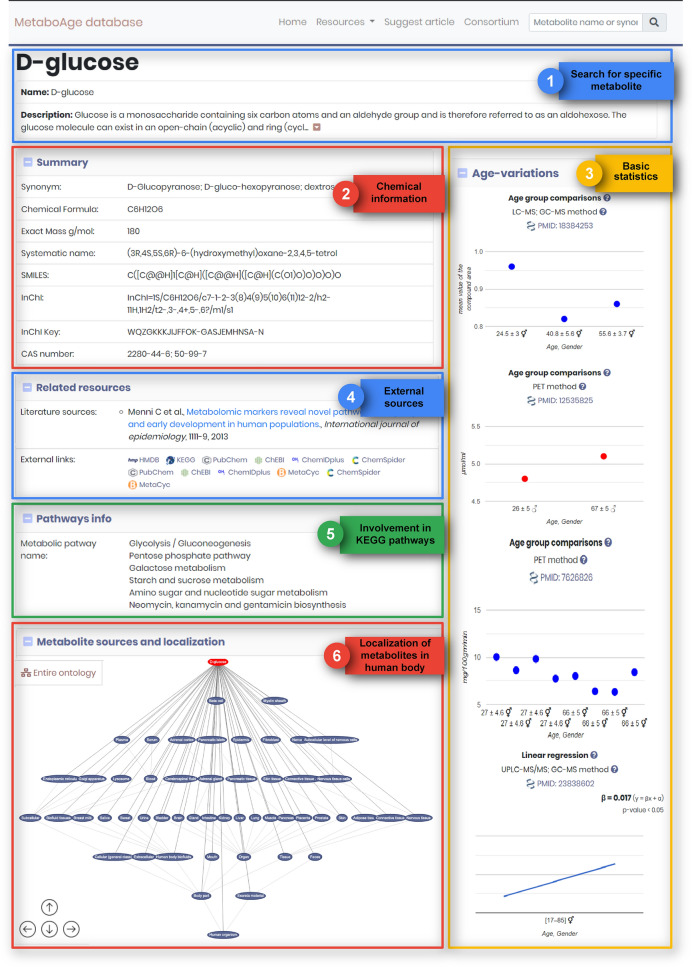
User guide. A short guideline on how the metabolite information is distributed on the website in various sections

Individual pages present metabolite-centric information, including a summary of biological and chemical information, metabolite variations (visually represented), related literature resources and external links, and metabolite sources and localization (Fig. 1).

The ‘Summary’ window contains general information on each metabolite, such as their approved synonyms, chemical formula, systematic name, SMILES (Simplified Molecular Input Line Entry System), InCHl, InCHl Key and CAS number, which are imported from PubChem based on their PubChem CID. In the ‘Related resources’ section are listed all major metabolomic databases in which the searched metabolite is annotated, together with their corresponding ID, providing users with cross-links to other databases, directly from the MetaboAge website. In this section, users can also find the literature sources based on which the metabolite was selected. The ‘Pathways info’ panel displays contents about the metabolic pathways in which the metabolites are involved, as well as their pathway map, imported from KEGG’s Ligand category of chemical information, KEGG Compound. Clicking on a pathway opens its corresponding entry on the KEGG website. The ‘Metabolite source and localization’ window displays the ontology of the searched metabolite, with its classes and subclasses. In this panel, users can visually navigate the metabolite-specific part of the ontology or can switch to the main (top-level) ontology view. By browsing MetaboAge directly through the location ontology (from its homepage), users can selectively access all metabolites expressed in various body parts or excreta materials.

In order to provide the user with a quick perspective on all age-related changes of a specific metabolite, individual pages also include an ‘Age-variations’ panel, in which gender-specific and method-specific metabolite variations are visually represented, grouped by the type of age-related variation into: ‘Age group comparisons’, ’Linear regression’ or ‘Log2(FC)’. These graphs display metabolite values for each age-groups, as well as the literature sources and the detection method used for measurements. Upon clicking on the button on the top-right corner of each metabolite’s entry page, the visual representation of the variations will be expanded, and complemented by a summary table, providing a detailed tabular overview of the information depicted in the graphs across studies. Lastly, to quickly familiarize the users with the database interface, the MetaboAge website includes a user guide under the “Resources” menu.

To provide programmatic access to the database, the MetaboAge website includes an API that allows querying the database. Users can request: (1) the metabolites included in the database (which will return a list of pairs ID/names), and (2) aging-related information on a specific metabolite, providing the metabolite ID and receiving a list of entries with metabolite variations. Each entry will include the metabolite ID, the method of detection, the mean value of the metabolite, beta value or log2 value, the standard deviation or the standard error, unit of measurement, the age group, the gender, the sample source, and the corresponding PubMed ID. For those metabolites that have more than one variation in the database, the output will consist of distinct lines with the aforementioned information, each line representing one variation. The data can be requested either in a JSON or API format.

## Materials and methods

### Scientific literature filtering and literature selection

In order to keep the database up-to-date and complete, scientific articles are continuously screened from PubMed. Using a set of general filters and keywords related to ageing and metabolomics, articles are automatically selected by custom-made Python/PostgreSQL scripts and their titles and abstracts are passed to data curators. Firstly, this automatic step ensures that studies related to diseases or clinical trials (which include keywords like “disease” or “medication”), are excluded from the list, as not being relevant for “healthy” ageing. Secondly, another filtering is carried out in order to determine with better accuracy those articles that contain information of interest for the database. By selecting articles which contain keywords such as “variation”, “longevity”, “ageing” or “metabolite”, we ensure the inclusion of most articles which contain data on metabolite variations with age. Under the section “Suggest article’, the website also allows users to help expand the database by suggesting articles with metabolomic changes that might be relevant to ageing. These suggestions will be considered by a curator, and upon revision, the MetaboAge team will decide whether to include the article as a source for extracting more metabolites and/or their variation with age.

Following the automatic screening, a final manual literature selection is done independently by at least two researchers, considering article titles and abstracts. Any disagreements are settled by reaching a consensus between the two reviewers or by discussing with a third person to avoid the erroneous exclusion of eligible articles. Whatever the case, records of why any article was excluded are kept internally.

### Standard operating procedure

To ensure the uniformity of the curation process by multiple curators we developed a Standard Operating Procedure (SOP) that contains detailed written instructions regarding the literature selection and database curation.

### Data curation

**Fig. 2:**
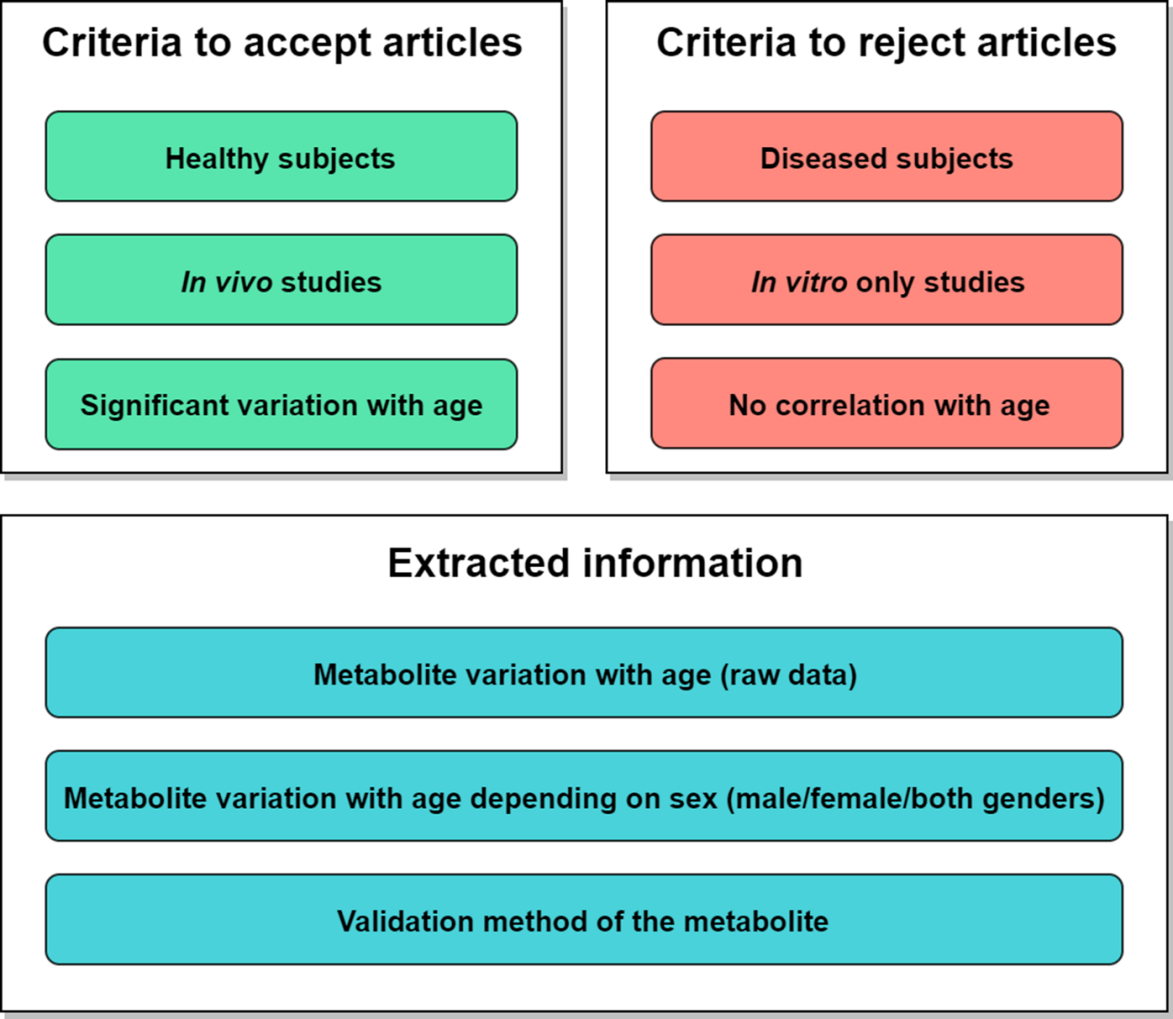
Article selection flowchart. Inclusion and exclusion criteria for scientific articles to be used as reports of metabolite variations for MetaboAge. From the accepted articles, all MetaboAge-relevant information is extracted according to the SOP

The articles selected for curation are then checked against established criteria as follows: (1) the included study has to show association of a human metabolite with ageing; (2) the metabolite variations or correlations with age have to be statistically significant; (3) in the published research, the determined metabolite concentration should not be altered by any supplements taken prior to or during the experiments; (4) the experimental subjects should not suffer from any medical condition and should not be under any medication during the experimental period. A simplified schematic of the inclusion/exclusion criteria and of the extracted data is shown in Fig. 2.

Upon passing the above criteria, metabolites and their age-related variation are extracted from the accepted articles and annotated in MetaboAge. The remaining articles selected from PubMed, which have not been selected after filters or manual curation, are briefly re-checked by the curation team, so as to make sure that all articles with relevant information are taken into account for the database.

During the curation process, if the enantiomer for a metabolite is not explicitly specified in the reviewed article, both enantiomers are considered and an attempt to infer the D/L relative configuration is performed.

If one of the enantiomers is not reported in established databases as a naturally-occurring isomer or if it is present only in nonhuman species, that configuration is disregarded. In the exceptional cases where both enantiomers are valid and the authors did not mention the used conformation, a note about this ambiguity is added to the metabolite description in MetaboAge.

Despite being used interchangeably in some other databases, in MetaboAge protonated and deprotonated forms of carboxylic acids are generally considered separate entities in order to unambiguously annotate metabolites. Additionally, when associating metabolites with KEGG pathways, a manual verification is performed to check whether the protonated or deprotonated compound is part of a specific pathway.

### Database ontology

**Fig. 3:**
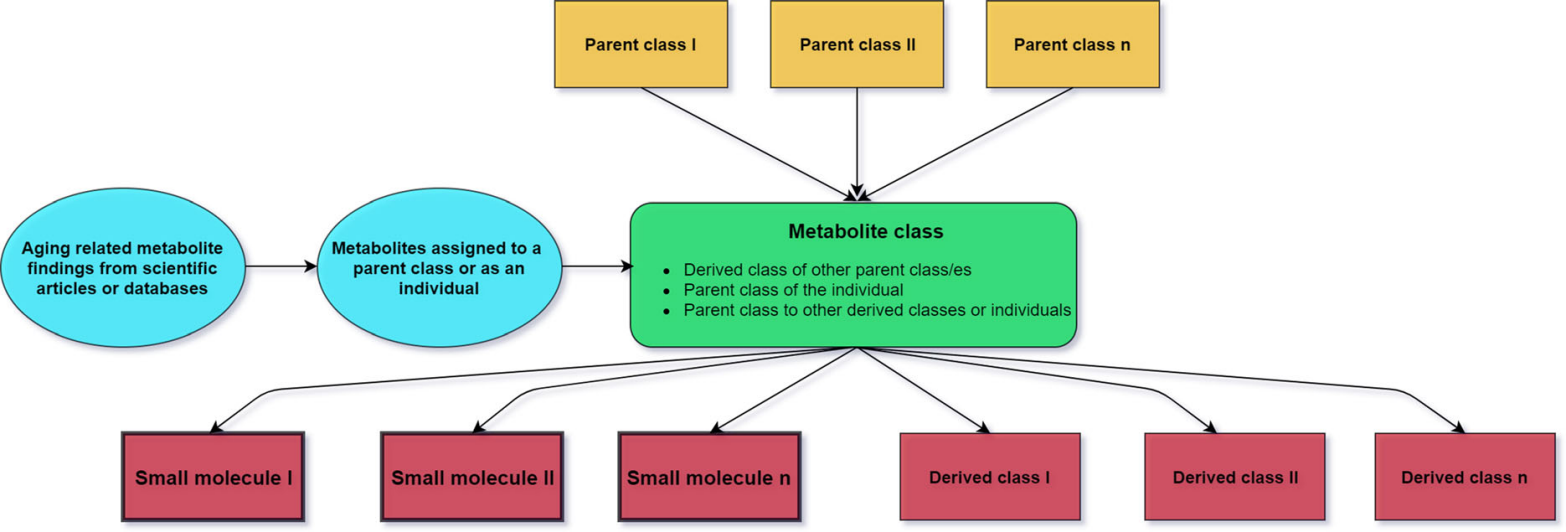
Metabolite assignment in the ontology classes. Every class is assigned to one or more parent classes and can be itself a parent class to one or more subclasses. Each class can contain any number of metabolites

The Protégé software (Version 4.3.0) was used to build a localization-based ontology for all metabolites in the database. Metabolites are represented in the ontology as “small molecules” that belong to one or more parent classes. At the same time, each class may have one or more derived classes, and may include any number of metabolites. Figure 3 gives a schematic representation of the metabolite localization-based ontology. Metabolites are assigned to metabolite classes based on their reported location or based on the source of the biological sample in which the measurement was carried out. Localizations are classified and sub-classed under principal classes which are as follows: human organism, body part, organ, tissue, cellular, subcellular and excreta material. Based on current data, MetaboAge defines a total of 111 metabolite classes. More information on subclasses and the number of metabolites in each class can be found in Supplementary Table S2.

## Conclusions

MetaboAge is a repository of metabolomic variations in healthy ageing providing quality data, which is manually curated through a well defined procedure. Additionally, the database integrates chemical annotations for molecular metabolomic entities based on information in well established resources, categorizes data based on an in-house constructed location-based ontology, and provides associations with metabolic pathways. Currently, MetaboAge contains more than 400 ageing-related metabolites and more than 1515 variations of these metabolites in different human age groups. By providing a large collection of ageing-related metabolite data, we aim for MetaboAge to support researchers conducting metabolomic studies of ageing, but also to serve as a broader tool for researchers working on a wide range of biological and health disciplines and metadata analysis.

## Electronic supplementary material

Below is the link to the electronic supplementary material.

Electronic supplementary material 1 List of databases used for metabolite annotations and the extracted information included in MetaboAge (DOCX 16 kb)

Electronic supplementary material 2 Metabolite number corresponding to the ontology classes in which they belong (DOCX 15 kb)

## Data Availability

MetaboAge is a publicly available database and can be freely accessed through its web interface. Currently, it is also possible to access the raw data from our database on request. Data will be made publicly available for download as soon as our article is published.
