# Supplementary material for: MetaboAge DB: a repository of known ageing-related changes in the human metabolome
Source: Biogerontology. 2020 Aug 12;21(6):763–71. doi: 10.1007/s10522-020-09892-w (PMC7541382; doi:10.1007/s10522-020-09892-w)
Supplement: Supplementary file 1 — Electronic supplementary material 1 List of databases used for metabolite annotations and the extracted information included in MetaboAge (DOCX 16 kb) [file 10522_2020_9892_MOESM1_ESM.docx]

**Supplementary Table 2:** Metabolite number corresponding to the ontology classes in which they belong

| **Metabolite localization**           **Number of metabolites**            **Number of subclasses** | | |
| --- | --- | --- |
| **Cellular (general class)** | | |
| Formative cells | 1 | 1 |
| Subcellular | 140 | 12 |
| **Extracellular** | | |
| Extracellular | 72 | 0 |
| **Human body biofluids** | | |
| Amniotic fluid | 1 | 0 |
| Bile | 3 | 0 |
| Biofluid tissues | 376 | 2 |
| Breast milk | 23 | 0 |
| Saliva | 86 | 0 |
| Seminal fluid | 3 | 0 |
| Sweat | 22 | 0 |
| Urine | 188 | 0 |
| **Mouth** | | |
| Mouth | 1 | 0 |
| **Organ** | | |
| Bladder | 19 | 0 |
| Brain | 32 | 9 |
| Eye | 2 | 1 |
| Gland | 31 | 3 |
| Heart | 4 | 0 |
| Intestine | 27 | 1 |
| Kidney | 55 | 0 |
| **Metabolite localization              Number of metabolites Number of subclasses** | | |
| **Organ** | | |
| Liver | 50 | 0 |
| Lung | 4 | 0 |
| Muscle | 32 | 3 |
| Pancreas | 16 | 1 |
| Placenta | 26 | 0 |
| Prostate | 49 | 0 |
| Skin | 41 | 1 |
| Spleen | 22 | 0 |
| **Spinal cord** | | |
| Spinal cord | 5 | 0 |
| **Tissue** | | |
| Adipose tissue | 24 | 0 |
| All tissues | 15 | 0 |
| Connective tissue | 44 | 2 |
| Hepatic tissue | 14 | 0 |
| Nervous tissue | 32 | 1 |
| **Feces** | | |
| Feces | 114 | 0 |
