# Supplementary material for: MetaboAge DB: a repository of known ageing-related changes in the human metabolome
Source: Biogerontology. 2020 Aug 12;21(6):763–71. doi: 10.1007/s10522-020-09892-w (PMC7541382; doi:10.1007/s10522-020-09892-w)
Supplement: Supplementary file 2 — Electronic supplementary material 2 Metabolite number corresponding to the ontology classes in which they belong (DOCX 15 kb) [file 10522_2020_9892_MOESM2_ESM.docx]

| **Database** | **Web access** | **Extracted information** |
| --- | --- | --- |
| PubChem | https://pubchem.ncbi.nlm.nih.gov/ | PubChem CID; synonyms; chemical formula; systematic name; SMILES; InChl; InChl Key; CAS number |
| ChemIDplus | https://chem.nlm.nih.gov/chemidplus/ | ChemIDplus ID |
| Chemical Entities of Biological Interest | https://www.ebi.ac.uk/chebi/ | ChEBI ID; description |
| NCI Thesaurus | https://ncit.nci.nih.gov/ncitbrowser/ | NCI thesaurus code; description |
| ChemSpider | http://www.chemspider.com/ | ChemSpider ID; description |
| Medical Subject Headings | https://www.ncbi.nlm.nih.gov/mesh | MeSH ID; description |
| Swiss Lipids | https://www.swisslipids.org/#/ | SwissLipids ID |
| Avanti Polar Lipids | https://avantilipids.com/ | Avanti Polar Lipids ID |
| FooDB | http://foodb.ca/ | FooDB ID; description |
| The Human Metabolome Database | http://www.hmdb.ca/ | HMDB ID; description; source; localization for onlotogy |
| METLIN | https://metlin.scripps.edu/ | METLIN ID |
| DRUGBANK | https://www.drugbank.ca/ | Drugbank ID |
| LIPID MAPS | https://www.lipidmaps.org/ | LIPID MAPS ID |
| MetaCyc | https://metacyc.org/ | MetaCyc ID |
| Japan Chemical Substance Dictionary | https://jglobal.jst.go.jp/en/ | NIKKAJI ID |
| Chemical Book | https://www.chemicalbook.com/ | CB ID |
| ChEMBL | https://www.ebi.ac.uk/chembl/ | ChEMBL ID |
| Protein Data Bank | https://www.rcsb.org/ | PDB ID |
| Kyoto Encyclopedia of Genes and Genomes | https://www.kegg.jp/kegg/kegg2.html | KEGG ID; pathway maps |
| 3DMET | http://www.3dmet.dna.affrc.go.jp/ | 3DMET ID |
| MetaboLights | https://www.ebi.ac.uk/metabolights/ | MetaboLights ID |
| KNApSAcK | http://www.knapsackfamily.com/KNApSAcK/ | KNApSAcK ID |
| BioCyc | https://biocyc.org/ | BioCyc ID |
| Biochemical Genetic and Genomic knowledge-base | http://systemsbiology.ucsd.edu/bigg-database | BiGG ID |
| Encyclopedia of Human Genes and Metabolism | https://humancyc.org/ | HumanCyc ID |

**Supplementary Table 1:** List of databases used for metabolite annotations and the extracted information included in MetaboAge
